# A Case of Anomalous Left Anterior Descending Artery Originating From the Right Sinus of Valsalva

**DOI:** 10.7759/cureus.15584

**Published:** 2021-06-10

**Authors:** Niravkumar Patel, Sukhdeep Bhogal, Vijay Ramu, Thomas Helton

**Affiliations:** 1 Internal Medicine, Johnston Memorial Hospital, Abingdon, USA; 2 Division of Cardiology, Department of Internal Medicine, East Tennessee State University, Johnson City, USA; 3 Division of Cardiology, James H. Quillen Veterans Affairs Medical Center, James H. Quillen College of Medicine, Johnson City, USA

**Keywords:** coronary artery, left anterior descending artery (lad), right coronary artery (rca), anomalous coronary artery, non-st segment elevation myocardial infarction (nstemi), right sinus of valsalva

## Abstract

The anomalous origin of coronary arteries has been extensively documented in the literature. Most of the anomalies are incidentally found either during coronary angiography or imaging studies and are usually benign; however, malignant outcomes have been reported in the literature. Here, we present the case of a 76-year-old male with non-ST segment elevation myocardial infarction who was found to have an asymptomatic anomalous origin left anterior descending artery from the right sinus of Valsalva.

## Introduction

The anomalous origin of coronary arteries has been extensively documented in the literature; however, the isolated coronary artery anomaly (CAA) is rare (0.6%-1.2%) [1-3]. Even rarer is the isolated anomalous origin of the left anterior descending artery (LAD) from the right sinus of Valsalva (RSV). Most of the anomalies are incidentally found during coronary angiography, coronary computed tomographic angiography, or coronary magnetic resonance angiography of coronary vessels. About 80% of the anomalies are benign and are asymptomatic; however, malignant outcomes have also been documented [4]. Here, we present the case of a 76-year-old male with non-ST segment elevation myocardial infarction (NSTEMI) who was found to have an asymptomatic anomalous origin of LAD from the RSV. To the best of our knowledge, only 67 such cases have been reported in the literature since the 1960s.

## Case presentation

A 76-year-old male presented to our hospital complaining of dyspnea on exertion and substernal chest pain radiating to the left arm that was relieved by nitroglycerine and rest. He had documented coronary artery disease with percutaneous coronary intervention (PCI) to the right coronary artery (RCA) and ramus intermedius (RI) done more than five years prior to presentation. Other medical history consisted of hypertension, dyslipidemia, chronic obstructive pulmonary disease, obstructive sleep apnea, and hepatitis C. He had a 40 pack-year smoking history but no illicit drug use history. Family history was negative for heart disease.

Physical exam showed an obese male with normal vital signs. He had a regular heart rate and rhythm without any murmur or jugular venous distension. Lungs were clear to auscultation and no evidence of peripheral edema or pulse deficit was noted in the extremities. The rest of the physical examination was largely unremarkable.

**Figure 1:**
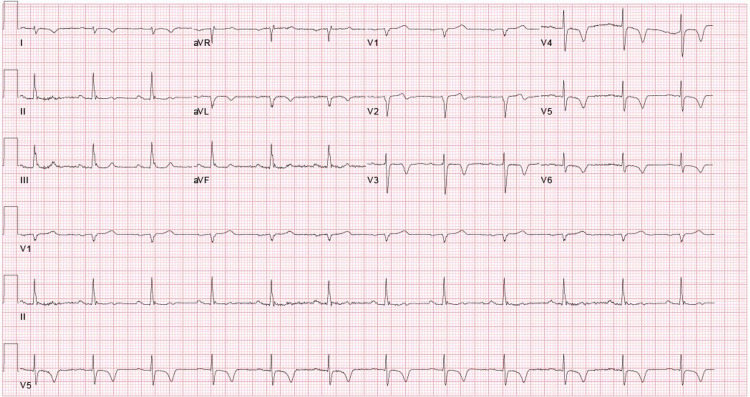
EKG showing sinus rhythm and anterolateral T-wave inversions

**Figure 2:**
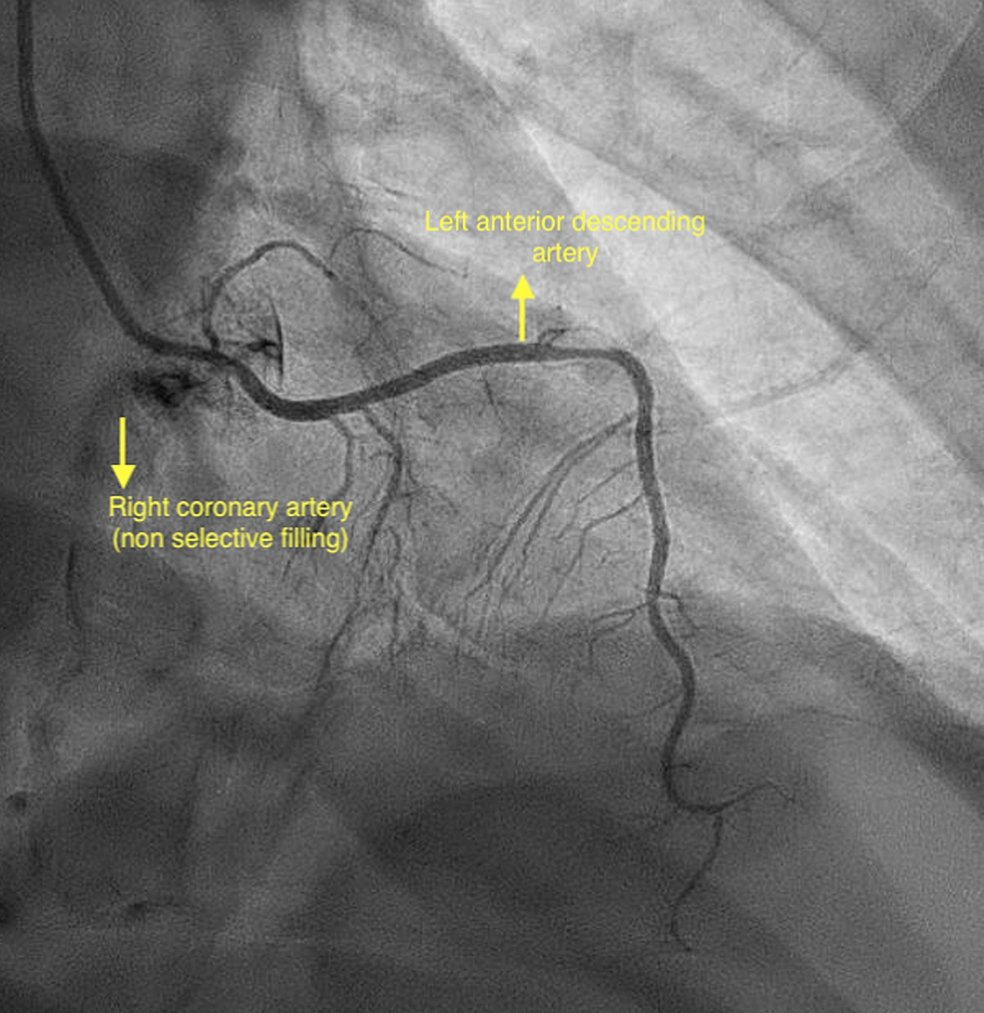
Right anterior oblique cranial view showing the left anterior descending artery arising from the right sinus of Valsalva with selective catheter engagement coursing left and upward before turning toward the apex

**Figure 3:**
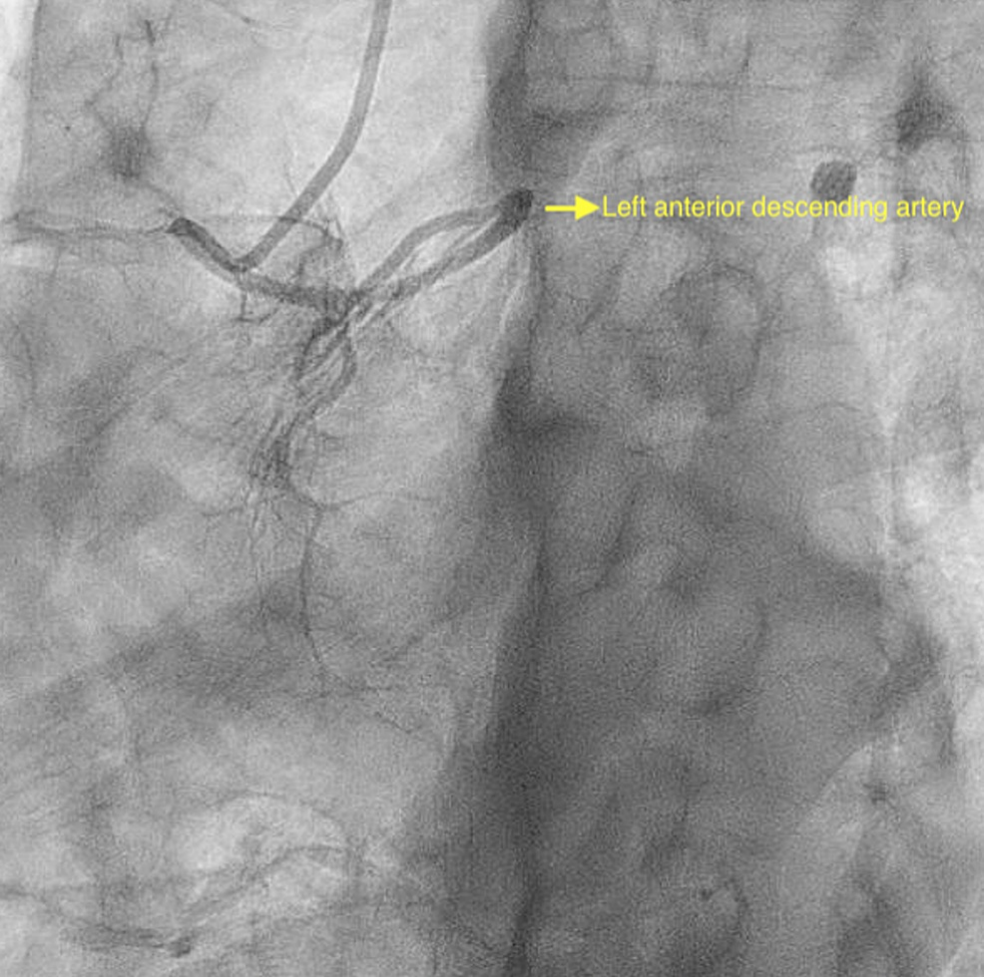
Left anterior oblique caudal view showing the anomalous origin of the left anterior descending artery from the right sinus of Valsalva with selective catheter engagement

**Figure 4:**
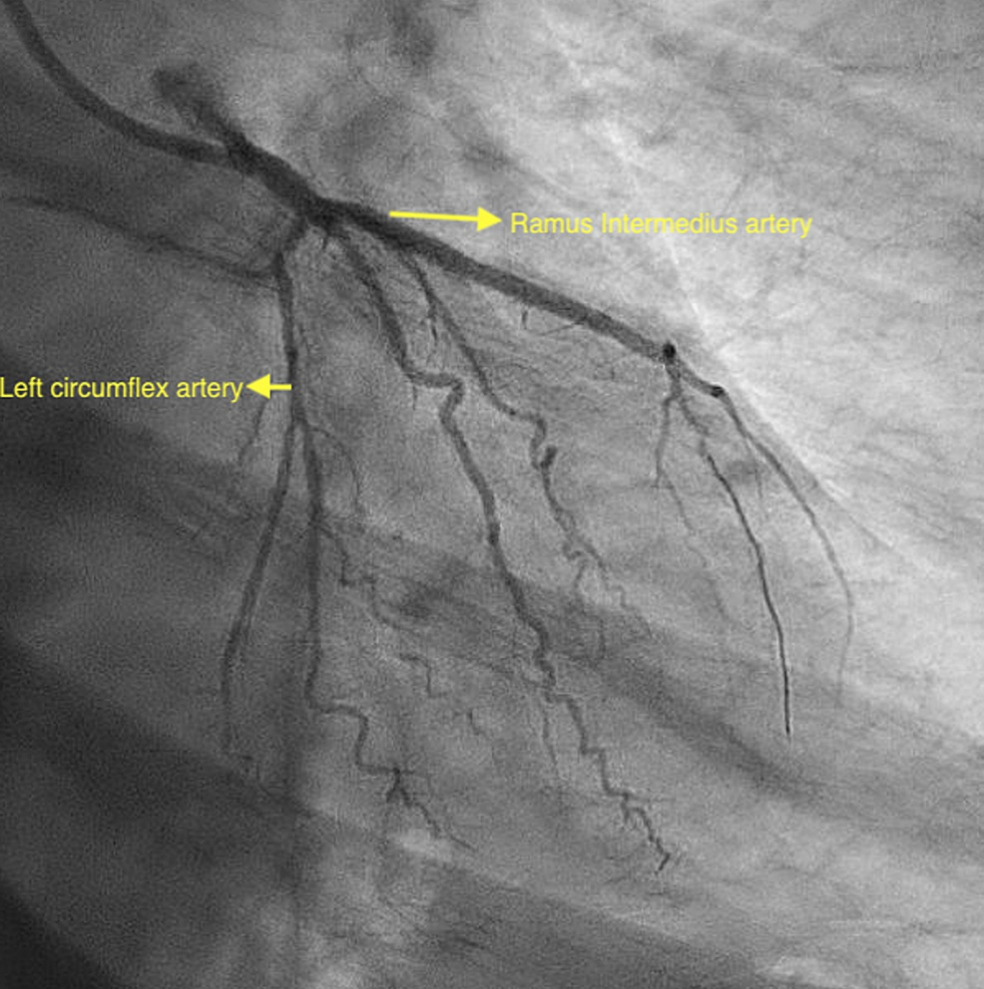
Right anterior oblique caudal view showing the missing left anterior descending artery and ramus intermedius post-percutaneous coronary intervention

Electrocardiogram (EKG) showed normal sinus rhythm with new T-wave inversions in anterolateral leads (Figure 1). Troponin I peaked at 40.2 ng/ml. He was given aspirin 325 mg and was started on a heparin drip. Bedside echocardiogram showed an ejection fraction of 40%-45% and moderate apical hypokinesis. A left heart catheterization was performed urgently showing in-stent thrombosis of a prior RI stent and an accidental finding of anomalous LAD originating from RSV instead of the left coronary cusp (Figures 2, 3). The patient subsequently underwent PCI of RI with a 2.5 x 38 mm drug-eluting stent (Figure 4). Non-obstructive disease was noted in other vessels. His postoperative course was uneventful and he was discharged on appropriate guideline-directed medical therapy.

## Discussion

CAAs are rare and the prevalence ranges from 0.21% to 5.79% [1-3,5]. The anomalies may differ depending upon their origin, course, and termination. They can be associated with other congenital cardiac anomalies such as tetralogy of Fallot, bicuspid aortic valve, transposition of great vessels, and arteriovenous fistulae [1,3,5]. Isolated anomalous LAD originating from RSV is an extremely rare coronary anomaly. Yamanaka and Hobbs in a review of 126,595 angiographic cases found 38 patients with this anomaly with an incidence of 0.03%, accounting for 2.3% of total coronary anomalies [3]. However, they did not discuss the clinical and angiographic features of these patients [6]. Two different retrospective reviews found 4 and 7 cases after reviewing 58,023 and 70,850 angiographies, respectively [6,7]. It typically takes an anterior free wall or a septal course, although retro-aortic and inter-arterial courses have been described in the literature [8,9]. Our patient had a benign anterior free wall course, in which LAD was seen passing to the left and upward before turning toward the apex in the right anterior oblique view (Figure 2) [9].

To the best of our knowledge, only 67 cases have been reported in the literature so far (Table 1). This anomaly should be differentiated from another anomaly in which LAD arises from the RCA, called as Type IV “dual LAD” anomaly, whose incidence is 0.01%-0.03% [10]. The majority of the CAAs are detected incidentally, and their clinical significance remains controversial. However, it has been postulated that an aberrant origin and the anomalous course could make them more prone to atherosclerosis [10]. Although CAAs are usually asymptomatic, they may present with angina, dyspnea, palpitations, syncope, cardiac arrest, and sudden cardiac death (SCD). According to Taylor et al., high-risk anatomy includes the course of the anomalous coronary artery between the aorta and pulmonary artery, and younger patients have a higher risk of SCD especially with exertional activities than the elderly [11]. CAAs are also the second most leading cause of SCD in athletes at 19% as compared to hypertrophic cardiomyopathy at 36% [12]. Other anatomic characteristics of CAAs predisposing them to grave outcomes include single coronary artery, origin from the pulmonary artery or opposite aortic sinus, intramural course, acute angle takeoff and ostial stenosis or atresia [2-4,6,10]. Asymptomatic CAAs rarely need interventions; however, symptomatic individuals with a malignant course often require surgical correction [8]. PCI of atherosclerotic lesion in anamalous coronary vessels could be challenging and is often limited by individual expertise [6].

**Table 1:** Summary of data on anomalous left anterior descending artery originating from the right coronary sinus

	Author	Number of cases	Number of total cases evaluated
1	Rajani et al. [13]	1	1
2	Contractor et al. [14]	1	1
3	Luebbering et al. [15]	1	1
4	Straalen et al. [16]	2	2
5	Fiorella et al. [17]	1	5
6	Tacar et al. [18]	1	1
7	Takenaka et al. [19]	1	1
8	Antonellis et al. [20]	1	1
10	Vicelj et al. [21]	1	1
11	Dalal et al. [22]	1	1
12	Yamanaka et al. [3]	38	126,595
13	Coyle and Thomas [8]	1	1
14	Harikrishnan et al. [23]	1	7400
15	Barriales et al. [24]	2	13,500
16	Sirasapalli et al. [25]	1	8021
17	Ghadri et al. [26]	2	11,541
18	Göl et al. [7]	4	58,023
19	Tuncer et al. [6]	7	70,850

Our patient had an anomalous origin of LAD from RSV; however, considering his age and no history of syncope and malignant arrhythmias, the course was likely benign and hence we opted to avoid any unnecessary imaging or intervention. He is currently on maximal medical therapy and has remained asymptomatic since the correction of the culprit lesion.

## Conclusions

CAAs are rare and usually asymptomatic; however, a high index of suspicion is required on part of clinicians about malignant characteristics that have the potential for lethal outcomes. Only a few cases of LAD from RSV have been documented in the literature. Our case highlights a benign course, but high-risk features should always be kept in mind.
